# A gene-targeted approach to investigate the intestinal butyrate-producing bacterial community

**DOI:** 10.1186/2049-2618-1-8

**Published:** 2013-03-04

**Authors:** Marius Vital, Christopher R Penton, Qiong Wang, Vincent B Young, Dion A Antonopoulos, Mitchell L Sogin, Hilary G Morrison, Laura Raffals, Eugene B Chang, Gary B Huffnagle, Thomas M Schmidt, James R Cole, James M Tiedje

**Affiliations:** 1 Center for Microbial Ecology, Michigan State University, East Lansing, MI, USA; 2 Department of Internal Medicine, University of Michigan, Ann Arbor, MI, USA; 3 Argonne National Laboratory, Argonne, IL, USA; 4 Marine Biological Laboratory, Woods Hole, MA, USA; 5 Knapp Center for Biomedical Discovery, University of Chicago, Chicago, IL, USA; 6 Department of Medicine, University of Chicago, Chicago, IL, USA

**Keywords:** Butyrate, Gene-targeted metagenomics, Human microbiome project, Pouchitis, Ulcerative colitis

## Abstract

**Background:**

Butyrate, which is produced by the human microbiome, is essential for a well-functioning colon. Bacteria that produce butyrate are phylogenetically diverse, which hinders their accurate detection based on conventional phylogenetic markers. As a result, reliable information on this important bacterial group is often lacking in microbiome research.

**Results:**

In this study we describe a gene-targeted approach for 454 pyrotag sequencing and quantitative polymerase chain reaction for the final genes in the two primary bacterial butyrate synthesis pathways, butyryl-CoA:acetate CoA-transferase (*but*) and butyrate kinase (*buk*). We monitored the establishment and early succession of butyrate-producing communities in four patients with ulcerative colitis who underwent a colectomy with ileal pouch anal anastomosis and compared it with three control samples from healthy colons. All patients established an abundant butyrate-producing community (approximately 5% to 26% of the total community) in the pouch within the 2-month study, but patterns were distinctive among individuals. Only one patient harbored a community profile similar to the healthy controls, in which there was a predominance of *but* genes that are similar to reference genes from *Acidaminococcus* sp., *Eubacterium* sp*., Faecalibacterium prausnitzii* and *Roseburia* sp., and an almost complete absence of *buk* genes. Two patients were greatly enriched in *buk* genes similar to those of *Clostridium butyricum* and *C. perfringens*, whereas a fourth patient displayed abundant communities containing both genes. Most butyrate producers identified in previous studies were detected and the general patterns of taxa found were supported by *16S rRNA* gene pyrotag analysis, but the gene-targeted approach provided more detail about the potential butyrate-producing members of the community.

**Conclusions:**

The presented approach provides quantitative and genotypic insights into butyrate-producing communities and facilitates a more specific functional characterization of the intestinal microbiome. Furthermore, our analysis refines *but* and *buk* reference annotations found in central databases.

## Background

The relationship between a healthy functioning gut microbiome and overall human well-being is firmly established. Recently, large-scale projects in this field, namely the Human Microbiome Project and the Metagenomics of the Human Intestinal Tract framework program, have been launched, with the goal of developing a holistic understanding of the composition and functional properties of intestinal bacteria and their effects on the human host. Numerous host-microbiome interactions have been reported and microbial-derived metabolites such as vitamins or short chain fatty acids have been of specific interest in many studies (see [1,2]). Among these, butyrate is considered as one of the most important metabolites as it serves as the major energy source of colonocytes; has anti-inflammatory properties; and regulates gene expression, differentiation and apoptosis in host cells [3].

Much of the information on the diversity of butyrate-producing bacteria has depended on culture-independent methods, however recent cultivation efforts for some of these strict anaerobes have been successful [4]. The existing isolates within this functional group are phylogenetically diverse, with the two most abundant groups related to *Eubacterium* spp. and *Roseburia* spp. (Clostridium cluster XIVa) and *Faecalibacterium prausnitzii* (Clostridium cluster IV) [5]. However, both clusters include additional non-butyrate-producing species. As such, *16S rRNA* gene-targeted analysis often cannot distinguish the butyrate-producing from the non-producing community in a sample [6]. Furthermore, it is increasingly recognized that horizontal gene transfer, which uncouples bacterial function from phylogeny, plays an important role in shaping the human microbiome [7]. The shortcomings of relying only on traditional *16S rRNA* gene-based phylogenetic analysis for functional inferences are now recognized in many other fields of microbial ecology. To resolve this, functional gene-targeted sequencing has emerged as the method of choice to investigate microbial functionality independent of phylogeny. This method has been used in several studies examining the nitrogen cycle [8], degradation of xenobiotic compounds [9] and antibiotic resistance of gut bacteria [10]. These studies have demonstrated the value of obtaining a detailed insight into specific microbial processes.

In the human gut, butyrate is produced through two main pathways, the butyryl-CoA:acetate CoA-transferase pathway (*but*) and the butyrate kinase (*buk*), and previous studies on colonic isolates of healthy individuals have illustrated that the *but* pathway predominates [11]. Consequently, Louis and Flint [12] developed a semi-quantitative PCR protocol targeting a selection of *but* sequences and used the same primers to construct clone libraries from fecal samples that revealed high gene diversity, including several unknown operational taxonomic units (based on a 98% DNA similarity [4]).

In this study, we present a novel approach that targets a broad range of *but* and *buk* genes based on both 454 pyrotag sequencing in combination with the Ribosomal Database Project’s (RDP) functional gene pipeline [13] and on quantitative PCR targeting selected groups of butyrate producers. The presented methods were applied on luminal samples from patients with ulcerative colitis (UC) who underwent a colectomy followed by ileal pouch anal anastomosis (IPAA) as described in the accompanying paper by Young *et al.*[14]. In this procedure, the entire colon is resected, the terminal ileum is fashioned into a pouch and connected to the anal canal, and intestinal flow is re-established. Previous data indicate that approximately half of patients will develop pouchitis within 1 year, an inflammatory condition similar to UC [15]. Because of the clinical similarity between pouchitis and UC, it is thought that studying the development of pouchitis can be used to reveal the etiology of UC. Several studies reported dysbiosis of the intestinal microbiome in patients with UC [16,17]. However, it is unclear whether the observed microbiome changes are the cause or the consequence of UC. These difficulties make pouchitis an ideal model system as it allows for the clinical observation of individuals from “time zero”, when fecal flow is initiated through the newly established, disease-free pouch. In this study, we specifically monitored the initial establishment (first 2 months) of butyrate-producing microbial communities in four patients after IPAA and compared the results with healthy controls.

## Methods

### Processing of samples

In this study, four patients with a history of UC undergoing total abdominal colectomy with IPAA were identified from the outpatient and inpatient practices of gastroenterologists and colorectal surgeons at the University of Chicago Medical Center between 2010 and 2011. All four patients had a confirmed diagnosis of UC based on endoscopy and pathology findings, were scheduled for a total proctocolectomy with IPAA, and were willing and able to participate in the study. Exclusion criteria included pregnancy or inability to give informed consent. All patients gave written informed consent before screening. The Institutional Review Board of the University of Chicago Medical Center approved this study protocol. For each patient, one sample was collected prior to ileostomy takedown (except for patient 200) and an additional three samples were collected over a period of 2 months after connection of the pouch to the anal canal (Table 1). None of the patients received antibiotic treatment during the period of this study. All samples were obtained from stool aspirates. Sterile saline was injected to liquefy the stool and contents were sampled using the suction port of the colonoscope. Bulk DNA was extracted using the UltraClean Mega Soil DNA Isolation Kit (MO BIO Laboratories, Inc., Carlsbad, CA, USA) according to the manufacturer’s protocol. Healthy colon samples were obtained from the recto-sigmoid section of the colon without prior bowel preparation to ensure that the microbiota was not altered by this procedure. For additional details on sample collection and storage, see [14].

**Table 1:** Samples analyzed in this study

	**Number of days from ileostomy takedown**			
**Patient**	**Visit 1**	**Visit 2**	**Visit 3**	**Visit 4**
200	−8	13	27	62
206	−1	18	32	60
207	−11	17	31	59
210	−2	19	33	61

### Primers, amplicon generation and 454 pyrotag sequencing

Primers were designed based on the Fungene database for the butyryl-CoA:acetate CoA-transferase (*but*) and butyrate kinase (*buk*) genes (Table 2 - for more details see Additional file 1: Figures S1 and S2 and Tables S1 and S2). Three barcoded forward and three reverse primers with fused adaptors for the Lib-A system (454 Life Sciences, Branford, CT, USA) were designed for each gene. The aim was to obtain broad coverage without exceeding a degeneracy of 100. For PCR, each forward primer (0.4 μM final concentration) was used separately in triplicate samples and was mixed with all three corresponding reverse primers (0.16 μM final concentration each), except for but_1F, where each reverse primer was used in a separate reaction. Because of the low target concentrations in many samples, sufficient amplification was often difficult. Therefore, extracted DNA was subjected to whole genome amplification (WGA; illustra GenomiPhi V2 DNA Amplification Kit, GE Healthcare, Little Chalfont, UK) to increase template concentration. A total of 150 ng of WGA template was used for each PCR reaction using the GoTaq Flexi system (Promega, Madison, WI, USA; total volume of 12.5 μL). Because primers do not perfectly match all desired targets (Additional file 1: Tables S1 and S2), PCR stringency was low for both genes with an annealing temperature of 54°C and high MgCl_2_ concentrations of 3 mM. Furthermore, higher cycle numbers (35×) were used to increase yield. Thermocycling was done as follows: 2 min at 95°C; 45 s at 95°C, 45 s at 54°C, 45 s at 72°C (×35); 10 min at 72°C. PCR products were pooled for each forward primer (triplicate reactions), gel-extracted (QIAquick Gel Extraction Kit; Qiagen, Valencia, CA, USA) and purified (QIAquick Gel Purification Kit; Qiagen). Several bands were visible on gels (especially for *but*) and only the target bands located around 425 (*but*) and 500 (*buk*) were excised. Nonspecific binding of primers was reduced with increased target concentrations. A re-conditioning step of purified product was essential to avoid short reads during sequencing. Each sample was re-amplified (0.2 ng of generated amplicons as template, 60°C annealing temperature, 15 cycles, total volume of 50 μL) using the AccuPrime PCR system (Life Technologies, Grand Island, NY, USA) with primers (0.4 μM final concentration) targeting whole adaptor sequences. PCR products were gel-extracted and purified again. Sequencing was performed with a 454 Junior System according to the manufacturer (454 Life Sciences). For each run, eight samples (four from each gene) were mixed at equal concentrations. We are aware that the protocol used, including WGA followed by a high cycle number PCR and a final re-amplification step, may have introduced bias. However, comparing all obtained results derived from different methods suggested that the procedure did not alter the main trends (see main text).

**Table 2:** Primers designed for this study are illustrated

**Functional genes - pyro-sequencing**			
**buk_1F**	atcaaYccDggWtcWacWtcWac	**buk_1R**	acHgcYttYtgRtttaaWgcatg
**buk_2F**	atWaatccWggttcWacWtcWacMaa	**buk_2R**	tgcYttYtggttgagygc
**buk_3F**	atMaaTccWggBtcKacMtcaact	**buk_3R**	gccttctgRttMagKgcatg
**but_1F**	cagctIggYatYggIgS	**but_1R**	aaRtccaIYtgIccVcc
**but_2F**	ggWatWggMgsYatgcc	**but_2R**	aaRtcaaSctgKccDc
**but_3F**	gHatYggIgStatgcc	**but_3R**	aagtcWaaYtgwccRcc
**Functional genes - quantitative PCR**			
**G_buk_F**	tgctgtWgttggWagaggYgga	**G_buk_R**	gcaacIgcYttttgatttaatgcatgg
**G_Acida_F**	cgcagaagaacattgacaagg	**G_Acida_R**	atggcagggttattgtctacataatc
**G_Fprsn_F**	gacaagggccgtcaggtcta	**G_Fprsn_R**	ggacaggcagatRaagctcttgc
**G_RosEub_F**	tcaaatcMggIgactgggtWga	**G_Ros_R**	tcgataccggacatatgccaKgag
		**G_Eub_R**	tcataaccgcccatatgccatgag
**16S genes - quantitative PCR**			
**Cbuty_F**	tactctgtaatggaggaagccact	**Cbuty_R**	ggtacaatgagatgcaacctcgc
**FPR-2F**^**a**^	ggaggaagaaggtcttcgg	**Fprau645R**^**a**^	Aattccgcctacctctgcact
**Rrec630F**^**a**^	cgKactagagtgtcggagg	**Erec870R**^**a**^	agtttYattcttgcgaacg
**RrecRi630F**^**a**^	gtcatctagagtgtcggagg		
**1132F**^**b**^	atggYtgtcgtcagctcgtg	**1108R**^**b**^	Gggttgcgctcgttgc

Degenerate bases are shown in capital letters. The following sequences are targeted: buk_F/R - butyrate kinase (*buk*) genes; but_F/R - butyryl-CoA:acetate CoA-transferase (*but*) genes; G_buk_F/R - *buk* genes of *Clostridium acetobutylicum*, *C. butyricum*, *C. perfringens*; G_Acida - *but* genes of *Acidaminococcus* sp.; G_Fprsn - *but* genes of *Faecalibacterium prausnitzii*; G_RosEub, G_Ros_R, G_Eub_R - *but* genes of *Eubacterium rectale* and *Roseburia* sp; Cbuty_F/R -16S genes of *C. butyricum*; FPR-2 F, Fprau645R - 16S genes of *F. prausnitzii*; Rrec630F, RrecRi630F, Erec870R - 16S genes of *E. rectale* and *Roseburia* sp.; 1132 F, 1108R - universal for 16S. ^a^ Primers described in Ramirez-Farias *et al.*[18]; ^b^ Primers described in Leigh *et al.*[19]. For more details on targeted sequences see Additional file 1: Table S1 and S2.

### Quantitative real time PCR

Primers designed for quantitative PCR (qPCR; Table 2) targeting the *but*/*buk* genes were based on the Fungene database and were specific to all desired target genes with at least two mismatches in one or both primers for other non-target *but*/*buk* genes. BLAST analysis illustrated no significant matches to other unrelated sequences. The *16S rRNA* gene primers (Rrec2 and Fprau) targeting butyrate producers are described in Ramirez-Farias *et al.*[18]. Total *16S rRNA* gene community qPCR primers were based on Leigh *et al.*[19]. Additionally, primers for the *16S rRNA* genes of *C. butyricum* were designed based on the RDP database. Specific amplification of targets was verified for all primers using the following pure cultures (amplification efficiency per nanogram of pure culture is given in brackets): *Bacillus licheniformis* ATCC 14580, *Bacteroidetes thetaiotaomicron* E50, *C. acetobutylicum* ATCC 824 (2.65 × 10^5^), *C. difficile* ATCC 630, *C. perfringens* ATCC 13124 (4.88 × 10^5^), *Eubacterium hallii* DSM(Z) 3353, *E. rectale* DSM(Z) 17629 (4.06 × 10^5^), *Faecalibacterium prausnitzii* DSM(Z) 17677 (5.53 × 10^5^), *Roseburia intestinalis* DSM(Z) 14610 (1.80 × 10^5^) and *R. inulinivorans* DSM(Z) 16841 (4.73 × 10^5^). Strains were purchased either from ATCC or DSM(Z) (as indicated in name). *B. licheniformi*s and *B*. *thetaiotaomicron* E50 were provided by Daniel Clemens. For the primers targeting *Acidaminococcus* (*but* gene) and *C. butyricum*, (*16S rRNA* gene), instead of a pure culture, a patient sample containing many target bacteria (based on all methods presented here) served as a positive control.

Amplification was performed with the SYBR Green Master Mix (Life Technologies) with 10 ng template DNA per reaction (total volume of 15 μL; no WGA except for the healthy control samples) in 384-well plates (ABI Prism 7900 HT, Life Technologies). Annealing temperatures and final primer concentrations were as follows: G_buk (64°C; 0.83 μM), Cbuty (66°C; 0.67 μM), FPR/Fprau (60°C; 0.83 μM), G_Acida (67°C; 0.83 μM), G_Fprsn (70°C; 0.83 μM), G_Ros/Eub (62°C; 0.83 μM; G_Ros_R and G_Eub_R were mixed together at equal final concentrations of 0.42 μM), Rrec/Erec (60°C; 0.83 μM; the two forward primers were mixed together at equal final concentrations of 0.42 μM) and total 16S (60°C; 0.67 μM). Thermocycling was done as follows; 2 min at 50°C; 10 min at 95°C; 45 s at 95°C; 45 s at individual annealing temperature; and 45 s at 72°C (for total *16S rRNA*, elongation at 72°C was omitted) (×40). Analysis was performed in duplicate samples. Genomic DNA of *R. inulinivorans*, *F. prausnitzii* and *C. perfringens* (for functional gene qPCRs) and cloned amplified products (for 16S qPCRs and G_Acida; TOPO cloning kit, Life Technologies) at concentrations of 10^2^ to 10^7^ copies (10-fold dilutions) were used for standard curves to determine target concentrations. Genomic DNA of *Desulfotomaculum acetoxidans* DSM 771 with 10 *16S rRNA* gene copy numbers was used for the standard curve (10^3^ to 10^8^) for total *16S rRNA* gene quantification. The detection limit was set as 10^2^ target sequences for all primers and results are expressed as a percentage of the total bacterial community based on total *16S rRNA* gene qPCR. For *16S rRNA* gene copy number normalizations of specific *16S rRNA* targets see below (comparing functional gene results to 16S pyrotag data). Because *but/buk* target sequences are present as a single copy per genome, qPCR results of functional genes were multiplied by five to account for multiple *16S rRNA* gene copies (five on average) of the intestinal bacterial flora).

### Sequence processing

Raw reads matching barcodes (106,708 for *but* and 84,222 for *buk*) were processed using the RDP pyro-sequencing pipeline [20], where 87% *but* and 94% *buk* sequences passed quality filtering. Subsequently, sequences were subjected to RDP FrameBot for frameshift corrections and closest match assignments. To develop a reference sequence set for FrameBot, we took the corresponding gene sequence sets from the Fungene database, developed through (Hidden Markov Model) HMM searches of the National Center for Biotechnology Information protein database, and removed partial sequences with less than 93% coverage (that is, last filled model position - first filled model position/model length) to the full gene length HMM model, giving 452 *but* and 422 *buk* reference sequences. For *buk*, 97% reads that passed the initial process passed FrameBot with minimum 30% identity to the closest match and 125 amino acids in length. On average, 1.6 frameshifts were corrected per sequence and 58% of the sequences contained at least one frameshift. For *but*, 59% reads that passed the initial process passed FrameBot with minimum 30% identity and 100 amino acids in length. The majority of non-passing sequences were identified as human origin. On average, 0.6 frameshifts were corrected per sequence, 30% of the sequences contained at least one frameshift. Sequences can be accessed at SRA062948.

### Ordination and diversity analysis

**Figure 1:**
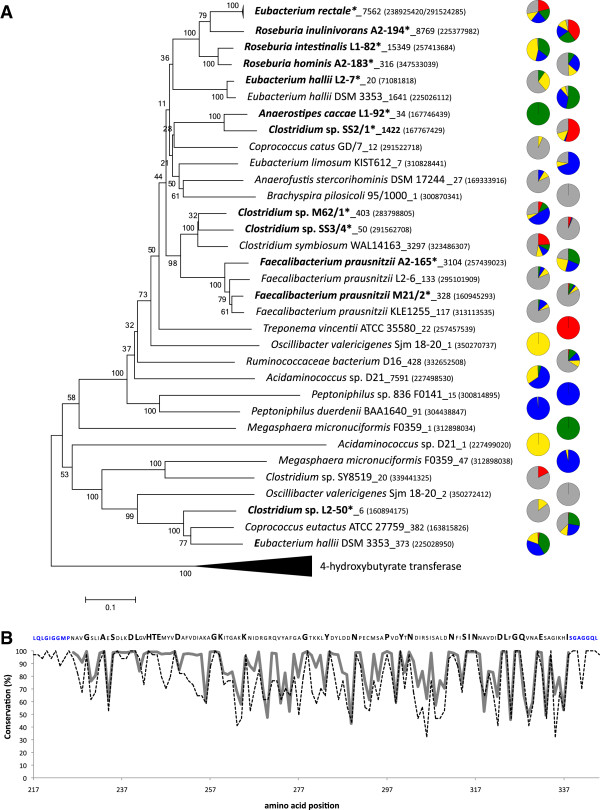
**Analysis of obtained butyryl-CoA:acetate CoA-transferase (*but*) sequences. (A)** Neighbor joining tree of all *but* reference sequences (closest hit from FrameBot) matching our amplicon data. Amount of amplicon sequences per closest match and reference sequence GI number (GenBank) are provided. Sequences marked with * are derived from bacteria with known *but* activity [5,11]. Pie charts illustrate the origin of amplicon sequences (red: patient 200, green: patient 206, blue: patient 207, yellow: patient 210 and grey: healthy controls). Note: relative abundance was investigated and the proportion of each color in the pie charts does not correspond to actual abundance of genes in samples. Relative community patterns per individual sample are presented in Additional file 1: Figure S5. Bootstrap values are indicated. **(B)** Conservation analysis of reference sequences from Panel **A** (dashed line) and of obtained amplicon sequences (thick grey line). The displayed sequence on top corresponds to the consensus sequence of all reference sequences. Bold amino acids demonstrate conserved sites (>95;%) in both reference and amplicon sequences. Blue amino acids on both the sequence ends illustrate primer binding sites.

**Figure 2:**
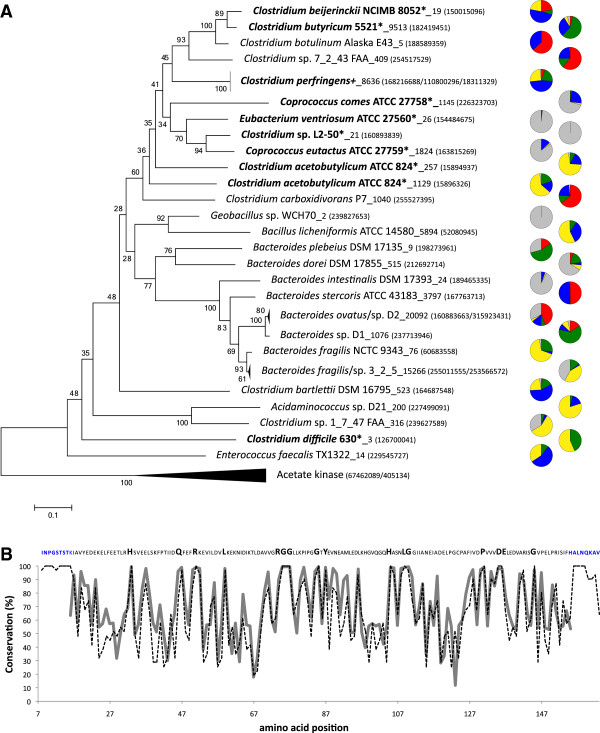
**Analysis of obtained butyrate kinase (*buk*) sequences. (A)** Neighbor joining tree of all *buk* reference sequences (closest hit from FrameBot) matching our amplicon data. Amount of amplicon sequences per closest match and reference sequence GI number (GenBank) are provided. Sequences marked with * or + highlight known butyrate producers [5,29]. Pie charts illustrate the origin of amplicon sequences (red: patient 200, green: patient 206, blue: patient 207, yellow: patient 210 and grey: healthy controls). Note: relative abundance was investigated and the proportion of each color in the pie charts does not correspond to actual abundance of genes in samples. Relative community patterns per individual sample are presented in Additional file 1: Figure S6. Bootstrap values are indicated. **(B)** Conservation analysis of reference sequences from Panel **A** (dashed line) and of obtained amplicon sequences (thick grey line). The displayed sequence on top corresponds to the consensus sequence of all reference sequences. Bold amino acids demonstrate conserved sites (>95%) in both reference and amplicon sequences. Blue amino acids on both sequence ends illustrate primer binding sites.

For each gene, the frameshift-corrected protein sequences were aligned using HMMER3 and clustered using RDP mcClust with the complete-linkage algorithm. Only amplicons with an identity of ≥70% to the closest matches in the reference (97% of *but* and 93% of *buk* sequences) were used for additional phylogenetic tree and ordination analysis, as we were not confident that more distant matches were bona fide *but* or *buk*. Additional filtering was performed based on neighbor joining tree analysis of reference sequences (see Results and Additional file 1). The remaining sequences were binned according to closest match assignments with reference sequences showing less than 2% dissimilarity merged (based on Figures 1 and 2). Results of both genes were combined and the entire butyrate community of each sample was used for ordination analysis. The nonmetric multidimensional scaling based on Chao corrected Jaccard index distance was performed using the vegan package in the R environment [21]. Both patients and time points were grouped for analysis. Diversity analysis (Shannon index) was calculated using the Biodiversity R package.

### Comparing functional gene results to 16S pyrotag data

For library generation of 16S rRNA gene analysis and pyro-sequencing see Young *et al.*[14]. For the first healthy control, no data on luminal aspirate were available and shown results are based on a colon biopsy sample of the same individual. Data were analyzed for known butyrate producers in the human colon at the genus level (based on [5] and obtained *but/buk* gene sequences) except for *Clostridia*, where species discrimination was applied. All results were normalized to five *16S rRNA* gene copy numbers, which represented the average for Firmicutes and Bacteroidetes, the two most abundant phyla in the gut. Average copy number of each genus was derived from rrnDB [22] and the Integrated Microbial Genome database [23]. A list of taxa searched as well as individual *16S rRNA* gene copy numbers is presented in Additional file 1: Table S3.

## Results

### Investigating *but* gene diversity

Several short chain fatty acid (SCFA) transferases have been characterized that exhibit broad substrate specificities and show remarkable sequence similarities [12]. Consequently, existing annotations in public databases are often unreliable and misleading. In our established Fungene database, many known *but* sequences are wrongly annotated (due to GenBank’s annotation) and SCFA transferases similar to *but* such as 4-hydroxybutyrate CoA transferases (*4hbt*) are present. A neighbor joining tree of all sequences from Fungene’s butyryl-CoA:acetate CoA-transferase (*but*) database (>93% coverage to model; to ensure only full-length sequences were considered) was constructed where all functionally verified *but* genes cluster together and apart from *4hbt* genes (Additional file 1: Figure S1). Primers were designed to specifically target those *but* sequences. However, it is still likely that SCFA transferases related to *but* are amplified as well*.* In order to quality filter our obtained *but* sequences (in addition to the processing pipeline presented in the Methods section)*,* only sequences located within the cluster identified in Additional file 1: Figure S1 were regarded as likely real *but*, whereas the remaining amplicons (<1%) matching 16 references outside the cluster were excluded from further analysis. We detected a broad diversity of *but* genes in our samples and they were linked to almost all described *but* carrying species (Figure 1A). Four closest FrameBot matches were assigned to 75% of all obtained sequences, namely *R. intestinalis* L1-82, *R. inulinivorans* A2-194, *Acidaminococcus sp.* D21 and *E. rectale* ATCC 33656. To verify the closest match assignments all amplicons were mapped on a tree together with full-length reference sequences using Pplacer ([24]; Additional file 1: Figure S3). We observed minimal deep branching; nearly all amplicons diverged in the terminal branches to the reference sequences, and the numbers assigned correlated well with the FrameBot closest match assignments. An exception was *Clostridium* sp. SS3/4 where many more amplicons than expected, that FrameBot had originally assigned to *C. symbiosum* and *Clostridium* sp. M62/1, mapped to that reference sequence. The discrepancies were most likely due to the different underlying assignment methods used by FrameBot and Pplacer. The former compares blossum62-corrected pairwise distances, whereas the latter is based on maximum likelihood criteria. Conservation analysis of *but* showed a remarkably similar pattern between the reference and amplicon sequences, and several well-conserved amino acid sites (>95% conservation in both groups) were identified (Figure 1B).

### Investigating *buk* gene diversity

A considerable diversity of *buk* sequences that included sequences similar to the majority of previously described butyrate producers were detected in our samples (Figure 2A). The Fungene database contains many sequences assigned to species not reported to produce butyrate, such as members of the phylum Bacteroidetes*.* Many of our amplicons closely matched sequences originating in *Bacteroides* and the established tree clusters them together with known butyrate producers and apart from acetate kinase, a closely related gene (Figure 2A; a neighbor joining tree of all Fungene sequences (93% cut-off) is shown in Additional file 1: Figure S2). Therefore, we included those sequences for analysis. Three quarters of all obtained *buk* amplicon sequences were assigned to four closest FrameBot matches; *Bacteroides* sp. D2, *Bacteroides* sp. 3_2_5, *C. butyricum* 5521 and *C. perfringens.* The resultant tree including the mapped amplicon sequences confirmed closest match assignments (Additional file 1: Figure S4). Sequence analysis revealed less similarity among *buk* genes than observed for *but* and fewer conserved amino acids could be detected (Figure 2B versus Figure 1B).

### Ordination and diversity analysis of obtained data

**Figure 3:**
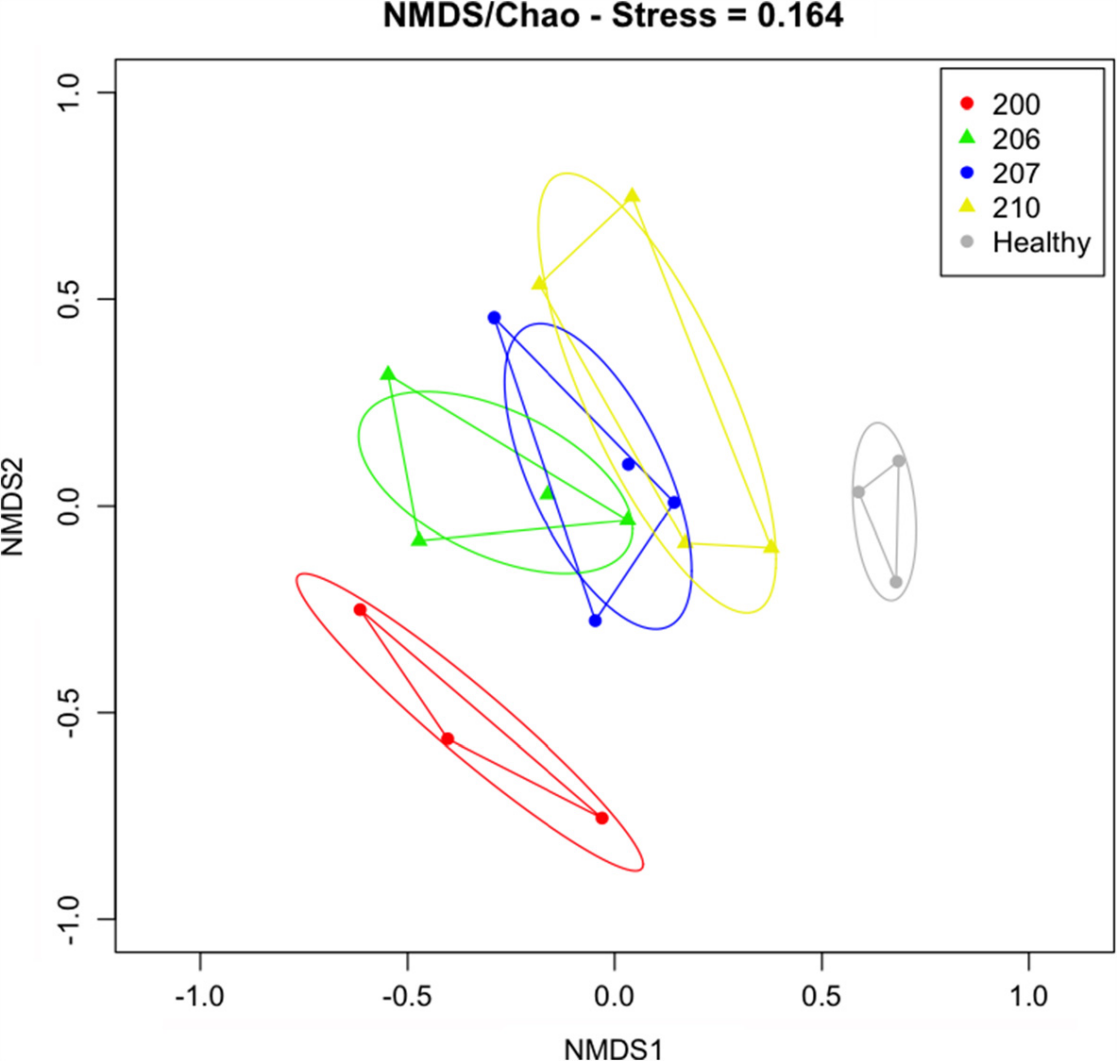
**Nonmetric multidimensional scaling analysis of the total butyrate-producing community - butyryl-CoA:acetate CoA-transferase (*but*) and butyrate kinase (*buk*) genes together - based on individual patients.** For explanation see text. Ellipses represent the 95% confidence interval on standard errors of means. Shepard plot for clustering is shown in Additional file 1: Figure S7.

**Figure 4:**
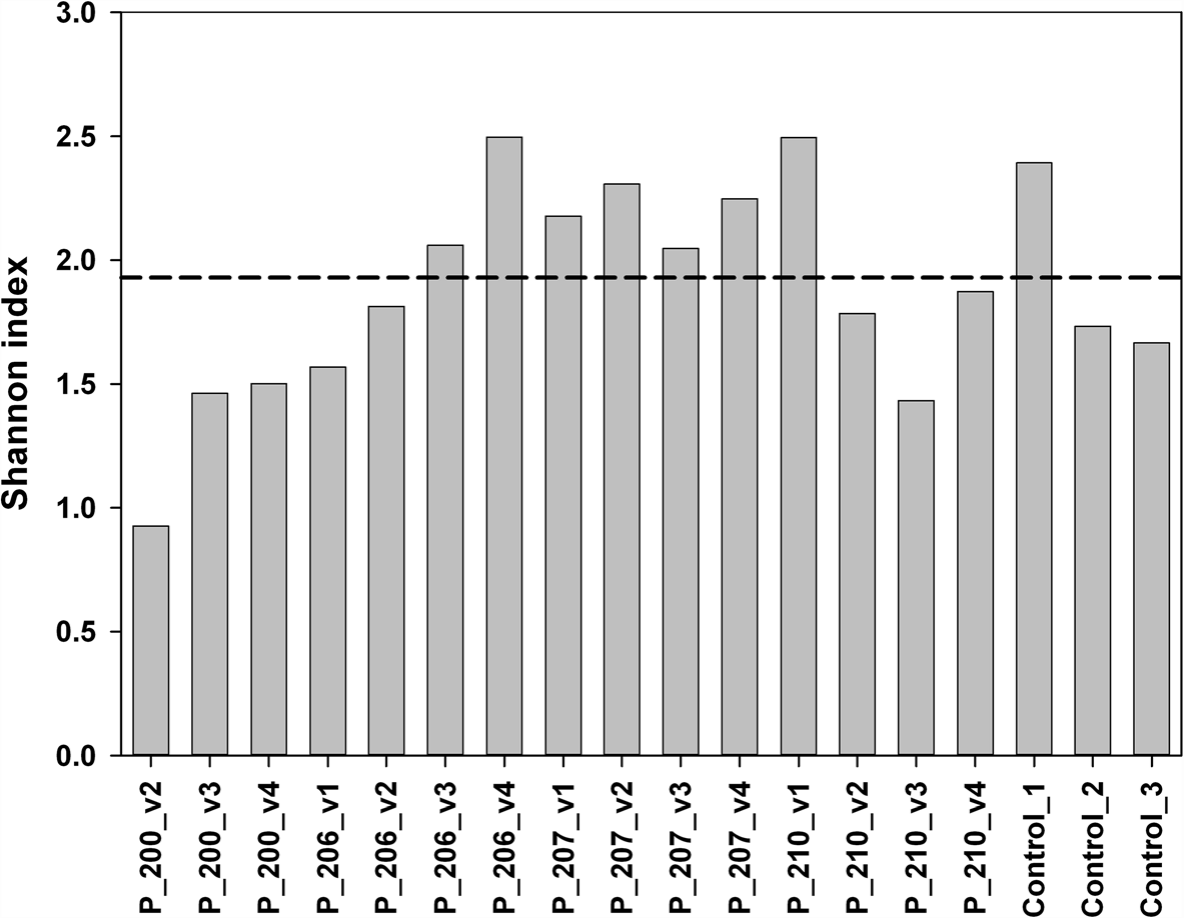
**Diversity analysis based on the Shannon index.** The dotted line illustrates the average value of the three healthy control samples.

Nonmetric multidimensional scaling analysis of the total butyrate-producing community (*but* and *buk* genes together) revealed a unique community pattern for individual patients, which all clustered distinct from the healthy control samples (Figure 3). However, the successional trend was different for all patients (Additional file 1: Figure S8). Diversity calculations also did not reveal a consistent successional pattern. Whereas Shannon diversity increased for patients 200 and 206, no change was detected for 207, and 210 demonstrated a decrease over time (Figure 4). At the fourth visit, all communities analyzed displayed a comparable diversity value, which was similar to that of the healthy control samples.

### Quantitative analysis of *but/buk* genes

**Figure 5:**
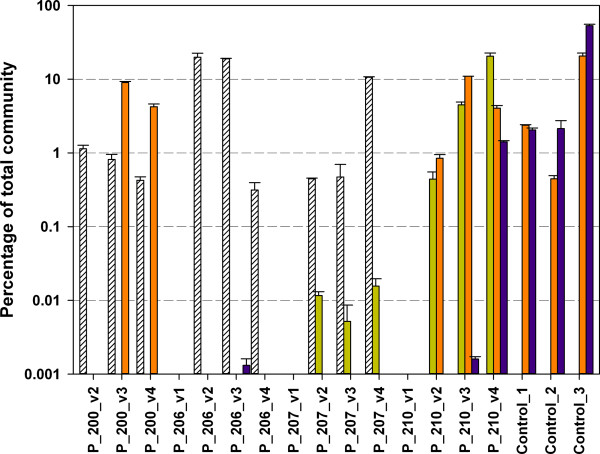
**Quantitative PCR analysis of butyryl-CoA:acetate CoA-transferase (*but*) and butyrate kinase (*buk*) genes.***But* of *Acidaminococcus* sp. (light green bars), *F. prausnitzii* (purple bars) and *Roseburia* sp. /*E. rectale* (orange bars) as well as *buk* linked to *C. butyricum, C. acetobutylicum* and *C. perfringens* (white coarse bars) were targeted. Percentage was calculated based on total *16S rRNA* gene qPCR data. Results were multiplied by five to account for multiple *16S rRNA* gene copy numbers of intestinal bacteria. The error bars represent the range on duplicate measurements.

Functional gene pyro-sequencing only allows for relative abundance measurements in each sample. Hence, we developed a complementary qPCR approach. Clear patterns emerged from qPCR of the *buk* and *but* genes. Whereas no target genes could be detected in the luminal aspirate before ileostomy takedown (visit 1; no sample was available for patient 200), abundant butyrate-producing communities became established in all patients over time (Figure 5). The observed community profiles were distinct between patients. Patients 206 and 207 were enriched in *buk* genes (up to 19.9% of the total community), whereas *but* was almost absent. Patient 210 was unique in the development of a community similar to the healthy controls, harboring *but* genes most closely related to both *F. prausnitzii* and *Roseburia* sp./*E. rectale*. Additionally, this patient exhibited abundant *but*-carrying *Acidaminococcus* sp. communities, which were absent in the healthy control samples. At visits three and four, 15.5% and 26% of patient 210’s total microbial community exhibited *but* genes, which was within the broad range for the control samples with 4.4%, 2.6% and 74.1%, respectively. In patient 200, we initially detected only *buk* genes, but a considerable *but*-gene community linked to *Roseburia* sp./*E. rectale* was established over time as well.

### Investigating the butyrate-producing community based on *16S rRNA* gene analysis

**Figure 6:**
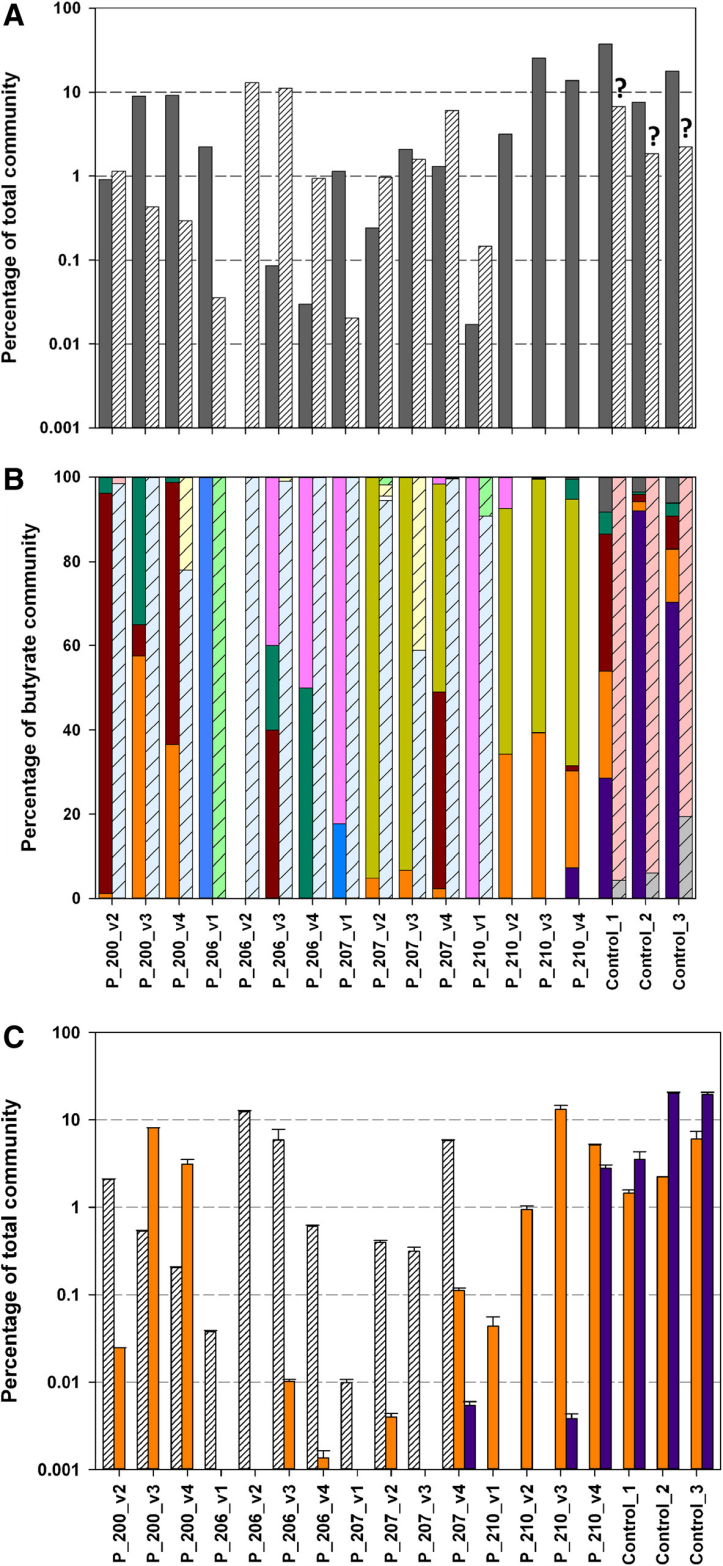
**Exploring the butyrate-producing bacterial community based on *16S rRNA *gene analysis. (A)** Candidates were split into butyryl-CoA:acetate CoA-transferase (*but*; grey bars) and butyrate kinase (*buk;* coarse white bars*)* containing groups. **(B)** Individual composition of *but* (*Acidaminococcus* sp. - olive, *Anaerostipes* sp. - dark green, *Coprococcus* sp. - dark red, *Eubacterium* sp. - black, *Faecalibacterium* sp. - dark purple, *Megasphaera* sp*.* - light purple, *Peptoniphilus* sp. - blue, *Oscillibacter* sp. - grey and *Roseburia* sp. - orange) and *buk* (*Anaerotruncus* - coarse grey, *C. beijerinckii -* coarse white, *C. butyricum -* coarse light blue, *C. perfringens* - coarse light yellow, *Enterococcus* sp. - light green and *Subdoligranulum* sp. - coarse light red) communities are given. **(C)** Quantitative PCR data targeting the 16S genes of *Faecalibacterium* sp. (purple bar), *Roseburia* sp./*E. rectale* (orange bar) and *C. butyricum* (white coarse bar). Note: *Coprococcus* sp. is considered to contain both *but* and *buk* genes. The error bars represent the range on duplicate measurements. All results are corrected for multiple *16S rRNA* copy numbers of individual bacteria (see Methods). ? - Butyrate production was shown for one strain of *Subdoligranulum* sp. and it is unclear whether all members of this genus have the ability to synthesize butyrate.

We retrieved the major known butyrate-producing taxa from literature [5] and from the *but* and *buk* data and used this information to screen for those taxa in the total 16S 454 pyrotag analysis presented in the accompanying paper [14]. Results are displayed in Figure 6A. Additionally, qPCR targeting specific butyrate producers was performed (Figure 6C). *16S rRNA* gene analysis supported the functional gene results in that similar overall patterns were detected by the two different techniques. Communities linked to *buk* were dominated by sequences similar to those of *C. butyricum* and *C. perfringens*, whereas sequences similar to *Acidaminococcus* sp., *F. prausnitzii* and *Roseburia* sp. comprised the majority of *but*-associated bacteria in both methods (Figures 5 and 6, Additional file 1: Figures S3 and S4). Nevertheless, several differences between *16S rRNA* gene and functional gene analysis were observed. Only a minute fraction from *16S rRNA* gene pyrotag data was identified as *Eubacterium* sp., whereas many *but* sequences were assigned to strains of *E. hallii* and *E. rectale*. Other studies that utilized fluorescence *in-situ* hybridization and clone libraries reported high concentrations of those strains in the healthy colonic microbial flora [4,25], which suggests that *16S rRNA* gene-based analysis could not reliably discriminate them from other taxa. Furthermore, *Subdoligranulum* sp., which contain one butyrate-producing isolate, *S. variabile* ([26] has the gene *buk*), were not detected in the functional gene data. But if this genus is considered to be butyrate-producing, then the *16S rRNA* gene analysis suggests a considerable abundance of *buk* genes in healthy control samples. Similarly, many more *16S rRNA* gene sequences were assigned to *Acidaminococcus* sp., *Anaerostipes* sp., C*oprococcus* sp. and *Peptoniphilus* sp. in certain samples compared with the results obtained from the functional gene analysis. These findings support earlier reports that butyrate synthesis is often not a homogenous feature of all members of a genus [4,5] and strengthens the application of higher taxonomic resolution techniques to adequately assess the butyrate-producing potential of bacterial communities. Species resolution is also crucial for the functionally diverse genus *Clostridia*. Several butyrate-producing members such as *Clostridium* sp. SS2/1, *Clostridium* sp. M62/1, *C. acetobutylicum, C. carboxidivorans* and *C. symbiosum* were matched to numerous functional gene sequences, but could not be detected in the *16S rRNA* gene data.

## Discussion

In this study we show that functional gene-targeted analysis of the intestinal bacterial butyrate-producing community can overcome limitations imposed by relying solely on *16S rRNA* gene targeted investigations. A combination of 454 pyrotag sequencing with qPCR analysis was essential to resolve the full differences among samples. Pyro-sequencing provided specific community profiles at great depth, whereas qPCR enabled the absolute quantification of genes. Ordination analysis based on pyrotag data revealed individual community patterns for each patient distinct from those of the healthy controls (Figure 3); however, only qPCR could demonstrate that overall gene concentrations differed over several orders of magnitude (Figure 5). Notably, the presented protocol for amplicon generation enabled amplification of genes for all samples, although actual abundance of individual targets was often below qPCR thresholds.

An abundant butyrate-producing community is essential for a well-functioning colon [27]. Butyrate is also the preferred energy generating substrate for the pouch epithelium and it is believed that supply deficiencies could initiate or promote development of pouchitis [28]. The pouch was aerobic before ileostomy takedown and only became anoxic after it was connected to the anal canal, which limits oxygen influx and promotes the establishment of anaerobes. Anoxic/oxic ratios of cultivars steadily increased over time after ileostomy takedown in all investigated patients [14]. In this study, we could demonstrate that these environmental changes were accompanied by the development of butyrate-producing communities at abundances similar to healthy participants of other studies [5] and to the healthy controls of this study. However, only patient 210 displayed a community pattern comparable to healthy control samples, which was also the case in the companion global 16S rRNA community analysis [14]. Patients 206 and 207 exhibited abnormal communities with *buk* genes predominating and only very few detectable *but* genes. Patient 200 displayed an ‘in-between’ community harboring both genes. Currently, the *buk* pathway is not considered to be important for butyrate production in healthy individuals [11], a finding further supported by this study. Whether the highly abundant *buk*-containing communities in patients 206 and 207 can compensate for low concentration of *but* is unclear. Unfortunately, no SCFA data are available to address this question. Enzyme assays on 17 butyrate-producing isolates demonstrated considerably higher activities for *but* than for *buk*[11], suggesting that the *but* pathway yields more butyrate in comparison to synthesis via *buk.* Interestingly, patient 210 is the only individual who did not show onset of inflammation 25 months post ileostomy takedown, whereas patients 200 (8 months), 206 (16 months) and 207 (17 months) all developed pouchitis. Although the patient number is low in this study, it does suggest that the initial establishment of a ‘healthy type’ butyrate-producing community is important to maintain a well-functioning pouch and to prevent the development of disease. The specific question of how butyrate production affects the development of disease will be addressed in a follow-up study where community profiles of patients undergoing IPAA will be monitored until the onset of inflammation and compared with those derived from asymptomatic individuals.

Our approach directly targets the genes coding for butyrate-synthesizing enzymes. We did observe some discrepancies between phylogeny and predicted function, which was especially true for the obtained *buk* gene sequences assigned to members of the genus *Bacteroides. Bacteroides* are currently not considered butyrate producers and several culture-based investigations point out their inability to synthesize butyrate (for examples, see [29,30]). This also applies to many other sequences presented in Additional file 1: Figure S2. Interestingly, some early studies from the 1980s indicated butyrate production by closely related bacteria, namely certain *Porphyromonas* (former *Bacteroides*) strains [31,32]. However, additional studies specifically investigating butyrate synthesis including more *Bacteroides* strains (and other candidates) under several different physiological conditions are needed to address this issue. Furthermore, even for known butyrate-synthesizing bacteria, gene detection does not automatically imply production of butyrate. Gene expression and a functioning pathway are determined by environmental conditions, with oxygen concentration as likely the most important factor [5]. Most butyrate producers are considered to be strict anaerobes with their growth and function strongly coupled. However, it has been recently shown that certain butyrate producers, namely *F. prausnitzii*, can also grow under microaerophilic conditions using extracellular oxygen as the final electron acceptor [33]. Butyrate production by this bacterium was still detected under these conditions but at a reduced rate.

## Conclusions

The presented protocols provide a new approach to more specifically resolve the butyrate-producing community. We could clearly demonstrate that butyrate producers were established at high abundance (approximately 5% to 26% of total bacterial community) in the pouch of all patients undergoing an IPAA within the first 2 months after ileostomy takedown. Community profiles were distinctive among patients. Most important, one individual harbored a community profile similar to the healthy controls with *but* genes predominating and an almost complete absence of *buk* genes, whereas the other three patients had other variants. Only the former patient remained healthy 25 months later. *16S rRNA* gene analysis showed similar overall patterns as the functional gene-targeted approach, but only the latter could reveal specific details on butyrate-producing taxa that were essential to assess the entire butyrogenic potential of the microbial communities analyzed. Furthermore, our analysis refines *but* and *buk* reference annotations found in central databases. In the near future, these methods will be complemented by metagenomic tools that will provide full-length gene sequences without prior amplification and will facilitate the investigation of not only individual genes of interest but also complete synthesis pathways.

## Abbreviations

BLAST: Basic Local Alignment Search Tool; buk: Butyrate kinase; but: Butyryl-CoA:acetate CoA-transferase; CoA: Coenzyme A; HMM: Hidden Markov Model; IPAA: Ileal pouch anal anastomosis; PCR: Polymerase chain reaction; qPCR: Quantitative polymerase chain reaction; RDP: Ribosomal Database Project; SCFA: Short chain fatty acid; UC: Ulcerative colitis; WGA: Whole genome amplification

## Competing interests

The authors declare that they have no competing interests.

## Authors’ contributions

All authors contributed in the organization and design of experiments as well as data interpretation and manuscript preparation. VBY, MLS, EBC, GBH, TMS and JMT developed the study. MV, CRP, QW, JRC and JMT wrote the paper. CRP and MV designed the primers. MV coordinated the laboratory work. QW, JRC and MV did sequence analysis and carried out the statistical analysis. LR, EBC and DAA provided the samples. MLS and HGM provided the 16S rRNA sequence data. All authors read and approved the final version of the manuscript.

## Supplementary Material

Additional file 1: Table S1 All sequences from the green highlighted section presented in **Figure S1** are shown (as they appear in the tree). Sequences with known butyryl-CoA:acetate CoA-transferase (*but*) activity [11] are shown in bold. Coverage of primers from this study (BUT_F/BUT_R) and of those presented in reference [12] (Ref_F/Ref_R) is shown, where number of mismatches (MM) per target sequence (based on RDP’s ProbeMatch) is indicated as a color code. BUT_F/BUT_R: green – 0-1 MM, yellow – 2 MM (results are merged from all 3 forward and reverse primers, respectively); Ref_F: green – 0-4 MM, yellow – 5 MM; Ref_R green - 0-2 MM, yellow – 3MM (categorization is based on primer description and testing from [12]). Sequences marked as red are predicted to not amplify. **Table S2.** All sequences from the green highlighted section presented in **Figure S2** are shown (as they appear in the tree). Sequences with known butyrate kinase (*buk*) activity [11] are shown in bold. Coverage of primers from this study (BUK_F/BUK_R) is shown, where number of mismatches (MM) per target sequence (based on RDP’s ProbeMatch) is indicated as a color code. Green – 0-1 MM, yellow – 2 MM (results are merged from all 3 forward and reverse primers, respectively). Sequences marked as red are predicted to not amplify. **Table S3.** Butyrate-producing candidates (based on [5] and additional taxa where *but/buk* genes were detected in this study) searched for in the obtained *16S rRNA* gene data and their corresponding gene copy numbers based on rrnDB (http://rrndb.mmg.msu.edu) and IMG (http://img.jgi.doe.gov). **Figure S1.** A neighbor joining tree of all sequences from Fungene’s butyryl-CoA:acetate CoA-transferase (*but*) database (>93% coverage to model; to ensure only full length sequences were considered). All *but* reference sequences with known function [10] group together in the section highlighted in green and apart from 4-hydroxybutyrate:butyryl CoA transferases (*4hbt*, highlighted in red). Several reference sequences from each group are indicated as stars (for *but* see **Table S1**, *4hbd*: *Clostridium klyuveri* (153955632), *C. tetani* (28210230), *Anaerostipes caccae* (76096774) and *C. aminobutyricum* (188032706)). All sequences in the green section are considered probable *but* sequences in this study. For details about primer coverage see **Table S1. Figure S2.** A neighbor-joining tree of all sequences from Fungene’s butyrate kinase (*buk*) database (>93% coverage to model; to ensure only full length sequences were considered). Eighty-eight percent of sequences are annotated as butyrate kinase and most sequences cluster apart from acetate kinase, a closely related gene (highlighted in red; two sequences with known acetate kinase function from *Bacillus subtilis* (405134) and *Escherichia coli* K-12 (67462089) were added to the tree (indicated as a star)). Only a few sequences have been verified biochemically as butyrate kinases ([10], indicated as stars) and all clustered together in one group (highlighted in green). Primers were designed to target most of the sequences in the green block as well as many targets outside this group. For details see **Table S2. Figure S3.** A maximum likelihood tree of FrameBot reference sequences for butyryl-CoA:acetate CoA-transferase (*but*) using PhyML [34]. Each amplicon sequence was placed onto this fixed reference tree using Pplacer [24] under maximum likelihood criteria. The height of each branch is proportional to the number of amplicons diverging from the tree along the branch. Bootstrap values are indicated. **Figure S4.** A maximum likelihood tree of FrameBot reference sequences for butyrate kinase (*buk*) using PhyML [34]. Each amplicon sequence was placed onto this fixed reference tree using Pplacer [24] under maximum likelihood criteria. The height of each branch is proportional to the number of amplicons diverging from the tree along the branch. Bootstrap values are indicated. **Figure S5.** Pyrosequencing results of amplified butyryl-CoA:acetate-CoA transferase (*but*) sequences. Results are shown as percentage (log10) of total reads per sample. **Figure S6.** Pyrosequencing results of amplified butyrate kinase (*buk*) sequences. Results are shown as percentage (log10) of total reads per sample. **Figure S7.** Shepard plot of the nonmetric multidimensional scaling (NMDS) analysis shown in Figure 3. **Figure S8.** Nonmetric multidimensional scaling (NMDS) analysis of the total butyrate producing community - butyryl-CoA:acetate-CoA transferase (*but*) *a*nd butyrate kinase *(buk)* genes together - based on visits is shown. Ellipses represent the 95% confidence interval on standard errors of means. Click here for file
