# Supplementary material for: A gene-targeted approach to investigate the intestinal butyrate-producing bacterial community
Source: Microbiome. 2013 Mar 4;1:8. doi: 10.1186/2049-2618-1-8 (PMC4126176; doi:10.1186/2049-2618-1-8)
Supplement: Additional file 1: Table S1 — All sequences from the green highlighted section presented in Figure S1 are shown (as they appear in the tree). Sequences with known butyryl-CoA:acetate CoA-transferase (but) activity [11] are shown in bold. Coverage of primers from this study (BUT_F/BUT_R) and of those presented in reference [12] (Ref_F/Ref_R) is shown, where number of mismatches (MM) per target sequence (based on RDP’s ProbeMatch) is indicated as a color code. BUT_F/BUT_R: green – 0-1 MM, yellow – 2 MM (results are merged from all 3 forward and reverse primers, respectively); Ref_F: green – 0-4 MM, yellow – 5 MM; Ref_R green - 0-2 MM, yellow – 3MM (categorization is based on primer description and testing from [12]). Sequences marked as red are predicted to not amplify. Table S2. All sequences from the green highlighted section presented in Figure S2 are shown (as they appear in the tree). Sequences with known butyrate kinase (buk) activity [11] are shown in bold. Coverage of primers from this study (BUK_F/BUK_R) is shown, where number of mismatches (MM) per target sequence (based on RDP’s ProbeMatch) is indicated as a color code. Green – 0-1 MM, yellow – 2 MM (results are merged from all 3 forward and reverse primers, respectively). Sequences marked as red are predicted to not amplify. Table S3. Butyrate-producing candidates (based on [5] and additional taxa where but/buk genes were detected in this study) searched for in the obtained 16S rRNA gene data and their corresponding gene copy numbers based on rrnDB (http://rrndb.mmg.msu.edu) and IMG (http://img.jgi.doe.gov). Figure S1. A neighbor joining tree of all sequences from Fungene’s butyryl-CoA:acetate CoA-transferase (but) database (>93% coverage to model; to ensure only full length sequences were considered). All but reference sequences with known function [10] group together in the section highlighted in green and apart from 4-hydroxybutyrate:butyryl CoA transferases (4hbt, highlighted in red). Several reference sequences from each group are [file 2049-2618-1-8-S1.pdf]

SUPPLEMENTAL INFORMATION

Table S1. All sequences from the green highlighted section presented in Figure S1 are shown (as they appear in the tree). Sequences with known butyryl-CoA:acetate CoA-transferase (*but*) activity [11] are shown in bold. Coverage of primers from this study (BUT_F/BUT_R) and of those presented in reference [12] (Ref_F/Ref_R) is shown, where number of mismatches (MM) per target sequence (based on RDP's ProbeMatch) is indicated as a color code. BUT_F/BUT_R: green – 0-1 MM, yellow – 2 MM (results are merged from all 3 forward and reverse primers, respectively); Ref_F: green – 0-4 MM, yellow – 5 MM; Ref_R green - 0-2 MM, yellow – 3MM (categorization is based on primer description and testing from [12]). Sequences marked as red are predicted to not amplify.

* Sequences indicated as stars in Figure S1.

GI	Target	BUT_F	BUT_R	Ref_F	Ref_R
**291524285**	**Eubacterium rectale DSM 17629**	green	green	green	green
**238925420**	**Eubacterium rectale ATCC 33656***	green	green	green	green
**291527689**	**Eubacterium rectale M104/1**	green	green	green	green
**225377982**	**Roseburia inulinivorans DSM 16841**	green	green	green	yellow
**257413684**	**Roseburia intestinalis L1-82**	green	green	green	green
**291534477**	**Roseburia intestinalis M50/1**	green	green	green	green
**347533039**	**Roseburia hominis A2-183***	green	green	green	green
**291518854**	**Butyrivibrio fibrisolvens 16/4**	green	green	green	green
257457539	Treponema vincentii ATCC 35580	green	green	red	red
320538370	Treponema phagedenis F0421	green	green	yellow	yellow
**291562708**	**Clostridium sp. SS3/4**	green	green	green	yellow
323486307	Clostridium symbiosum WAL-14163	green	green	red	red
**283798805**	**Clostridium sp. M62/1**	green	green	green	green
295092701	Clostridium cf. saccharolyticum K10	green	green	green	green
**257439023**	**Faecalibacterium prausnitzii A2-165***	green	green	green	green
**295101909**	**Faecalibacterium prausnitzii L2-6**	green	green	green	yellow
313113535	Faecalibacterium cf. prausnitzii KLE1255	green	green	green	yellow
**160945293**	**Faecalibacterium prausnitzii M21/2**	green	green	green	green
**295105498**	**Faecalibacterium prausnitzii SL3/3**	green	green	green	yellow
**71081818**	**Eubacterium hallii***	green	green	green	green
225026112	Eubacterium hallii DSM 3353	green	green	green	green
169333916	Anaerofustis stercorihominis DSM 17244	green	green	red	red
291522718	Coprococcus catus GD/7	green	green	green	yellow
**167767429**	**Clostridium sp. SS2/1**	green	green	green	green
**167746439**	**Anaerostipes caccae DSM 14662**	green	green	green	green
317471244	Anaerostipes sp. 3_2_56FAA	green	green	green	green
306820361	Eubacterium yurii subsp. margaretiae ATCC 43715	green	green	red	red
310828441	Eubacterium limosum KIST612	green	green	green	green
300870341	Brachyspira pilosicoli 95/1000	green	green	red	red
296127049	Brachyspira murdochii DSM 12563	green	green	red	red
225621129	Brachyspira hyodysenteriae WA1	green	green	red	red
343387568	Brachyspira intermedia PWS/A	green	green	red	red
350270737	Oscillibacter valericigenes Sjm18-20	green	green	red	red
313904206	Eubacterium cellulosolvens 6	green	green	green	yellow
340749848	Fusobacterium mortiferum ATCC 9817	green	green	red	red
299143268	Peptoniphilus sp. oral taxon 386 str. F0131	green	green	red	red

350567088	Peptoniphilus indolicus ATCC 29427	
282882597	Peptoniphilus lacrimalis 315-B	
300814895	Peptoniphilus sp. oral taxon 836 str. F0141	
304438847	Peptoniphilus duerdenii ATCC BAA-1640	
313888015	Peptoniphilus harei ACS-146-V-Sch2b	
169333899	Anaerofustis stercorihominis DSM 17244	
227498530	Acidaminococcus sp. D21	
332652508	Ruminococcaceae bacterium D16	
77920041	Pelobacter carbinolicus DSM 2380	
297617068	Syntrophothermus lipocalidus DSM 12680	
256544590	Anaerococcus vaginalis ATCC 51170	
212697489	Anaerococcus hydrogenalis DSM 7454	
325847081	Anaerococcus hydrogenalis ACS-025-V-Sch4	
227485260	Anaerococcus lactolyticus ATCC 51172	
227501351	Anaerococcus tetradius ATCC 35098	
257066838	Anaerococcus prevotii DSM 20548	
325479646	Anaerococcus prevotii ACS-065-V-Col13	
114567446	Syntrophomonas wolfei subsp. wolfei str. Goettingen	
167629587	Heliobacterium modesticaldum Ice1	
114565991	Syntrophomonas wolfei subsp. wolfei str. Goettingen	
114566545	Syntrophomonas wolfei subsp. wolfei str. Goettingen	
315926008	Pseudoramibacter alactolyticus ATCC 23263	
154496087	Pseudoflavonifractor capillosus ATCC 29799	
89896112	Desulfitobacterium hafniense Y51	
354559742	Desulfitobacterium metallireducens DSM 15288	
345860185	Desulfosporosinus sp. OT	
354563995	Desulfosporosinus meridiei DSM 13257	
333977817	Desulfotomaculum kuznetsovii DSM 6115	
89901049	Rhodoferax ferrireducens T118	
114566674	Syntrophomonas wolfei subsp. wolfei str. Goettingen	
297616690	Syntrophothermus lipocalidus DSM 12680	
114565869	Syntrophomonas wolfei subsp. wolfei str. Goettingen	
114566325	Syntrophomonas wolfei subsp. wolfei str. Goettingen	
224368716	Desulfobacterium autotrophicum HRM2	
134298229	Desulfotomaculum reducens MI-1	
134299348	Desulfotomaculum reducens MI-1	
355358595	Desulfotomaculum gibsoniae DSM 7213	
134299639	Desulfotomaculum reducens MI-1	
258515366	Desulfotomaculum acetoxidans DSM 771	
89894457	Desulfitobacterium hafniense Y51	
219668882	Desulfitobacterium hafniense DCB-2	
354557839	Desulfitobacterium metallireducens DSM 15288	
354559612	Desulfitobacterium metallireducens DSM 15288	
134298259	Desulfotomaculum reducens MI-1	
302344690	Desulfarculus baarsii DSM 2075	
302344691	Desulfarculus baarsii DSM 2075	
290968082	Megasphaera genomosp. type_1 str. 28L	
348025676	Megasphaera elsdenii DSM 20460	
124263109	Methylibium petroleiphilum PM1	
11498745	Archaeoglobus fulgidus DSM 4304	
11499439	Archaeoglobus fulgidus DSM 4304	
312898034	Megasphaera micronuciformis F0359	
342218086	Megasphaera sp. UPII 135-E	
348025661	Megasphaera elsdenii DSM 20460	
282856351	Pyramidobacter piscolens W5455	
313906028	Eubacterium cellulosolvens 6	
350272412	Oscillibacter valericigenes Sjm18-20	
**160894175**	**Clostridium sp. L2-50***	
225028950	Eubacterium hallii DSM 3353	
163815826	Coprococcus eutactus ATCC 27759	
**295093958**	**Coprococcus sp. ART55/1***	
312898038	Megasphaera micronuciformis F0359	
339441325	Clostridium sp. SY8519	
342218651	Megasphaera sp. UPII 135-E	
290968029	Megasphaera genomosp. type_1 str. 28L	
335049424	Megasphaera sp. UPII 199-6	
**291561529**	**Clostridium sp. SS3/4**	
227499020	Acidaminococcus sp. D21	
284047465	Acidaminococcus fermentans DSM 20731	
332652648	Ruminococcaceae bacterium D16	
**291087341**	**Clostridium sp. M62/1**	
295089819	Clostridium cf. saccharolyticum K10	
323694561	Clostridium symbiosum WAL-14673	
323487052	Clostridium symbiosum WAL-14163	
355623373	Clostridium sp. 7_3_54FAA	

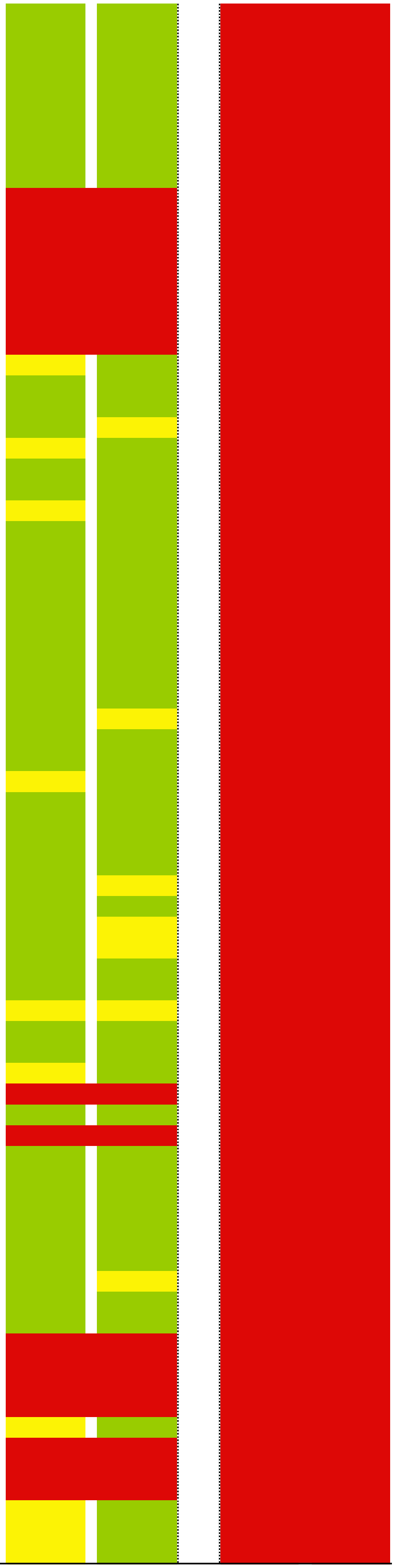

Table S2. All sequences from the green highlighted section presented in Figure S2 are shown (as they appear in the tree). Sequences with known butyrate kinase (*buk*) activity [11] are shown in bold. Coverage of primers from this study (BUK_F/BUK_R) is shown, where number of mismatches (MM) per target sequence (based on RDP's ProbeMatch) is indicated as a color code. Green – 0-1 MM, yellow – 2 MM (results are merged from all 3 forward and reverse primers, respectively). Sequences marked as red are predicted to not amplify.

GI	Target	BUK_F	BUK_R
168178887	Clostridium botulinum NCTC 2916	red	red
153940045	Clostridium botulinum F str. Langeland	red	red
170757474	Clostridium botulinum B1 str. Okra	red	red
148381366	Clostridium botulinum A str. ATCC 3502	red	red
187776630	Clostridium sporogenes ATCC 15579	green	yellow
168181920	Clostridium botulinum Bf	red	red
170759961	Clostridium botulinum A3 str. Loch Maree	red	red
255527395	Clostridium carboxidivorans P7	green	yellow
110802483	Clostridium perfringens SM101	green	green
110800296	Clostridium perfringens ATCC 13124	green	green
18311329	Clostridium perfringens str. 13	green	green
168216688	Clostridium perfringens NCTC 8239	green	green
28212124	Clostridium tetani E88	red	red
457634	Clostridium acetobutylicum	green	green
**15896326**	**Clostridium acetobutylicum ATCC 824***	green	green
**150019419**	**Clostridium beijerinckii NCIMB 8052***	yellow	yellow
**150018818**	**Clostridium beijerinckii NCIMB 8052**	green	green
**15894937**	**Clostridium acetobutylicum ATCC 824**	green	green
302876475	Clostridium cellulovorans 743B	green	yellow
254517530	Clostridium sp. 7_2_43FAA	green	green
254517529	Clostridium sp. 7_2_43FAA	green	green
**150015096**	**Clostridium beijerinckii NCIMB 8052**	green	green
182419451	Clostridium butyricum 5521	green	green
187932621	Clostridium botulinum B str. Eklund 17B	green	green
251779072	Clostridium botulinum E1 str. 'BoNT E Beluga'	green	green
188589359	Clostridium botulinum E3 str. Alaska E43	green	green
**295094051**	**Coprococcus sp. ART55/1***	green	yellow
163815269	Coprococcus eutactus ATCC 27759	green	green
**160893839**	**Clostridium sp. L2-50***	green	green
154484675	Eubacterium ventriosum ATCC 27560	green	green
260437207	Butyrivibrio crossotus DSM 2876	green	green
261366431	Subdoligranulum variabile DSM 15176	red	red
336429378	Lachnospiraceae bacterium 3_1_57FAA_CT1	red	red
	**92 targets outside cluster**		

Table S3. Butyrate-producing candidates (based on [5] and additional taxa where *but/buk* genes were detected in this study) searched for in the obtained *16S rRNA* gene data and their corresponding gene copy numbers based on rrnDB (http://rrndb.mmg.msu.edu) and IMG (http://img.jgi.doe.gov).

**Names**	***Terminal gene***	***16S rRNA* copy number**
Acidaminococcus sp.	*but*	5
Anaerofustis sp.	*but*	2
Anaerotruncus sp.	*buk*	4
Brachysoira sp.	*but*	?
Butyrivibrio sp.	*but*	6
Clostridium acetobutylicum	*buk*	11
C. beijerinckii	*buk*	14
C. barlettii	*buk*	?
C. botulinum	*buk*	9
C. butyricum	*buk*	18
C. carboxidivorans	*buk*	13
C. difficile	*buk*	10
Clostridium sp. GM2/1	*but*	?
Clostridium sp. M62/1	*but*	1
Clostridium sp. SS2/1	*but*	12
Clostridium sp. SS3/4	*but*	?
Clostridium sp. SSC2	*but*	1
Clostridium sp. SY8519	*but*	4
Clostridium sp. 1_7_47 FAA	*buk*	1
Clostridium sp. 7_2_43 FAA	*buk*	1
C. perfringens	*buk*	10
C. sporogenes	*buk*	8
C. symbiosum	*but*	1
C. tetani	*buk*	6
Coprococcus sp.	*but / buk*	3
Enterococcus sp.	*buk*	5
Eubacterium sp.	*but*	5
Faecalibacterium sp.	*but*	5
Megasphaera sp.	*but*	2
Oscillibacter sp.	*but*	3
Peptoniphilus sp.	*but*	1
Treponema	*but*	2
Subdoligranulum sp.	*buk*	1

? – no data available.

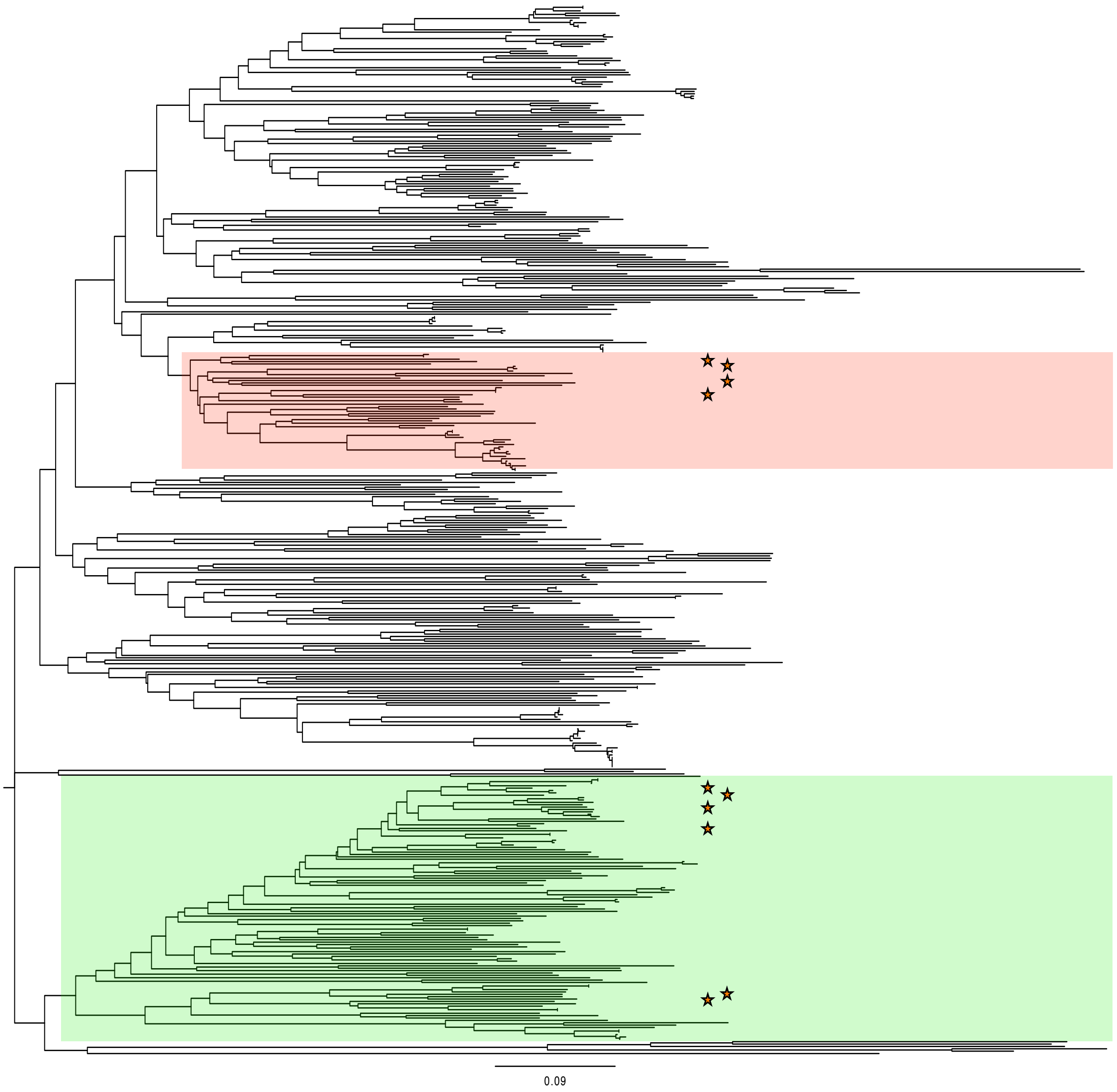

Figure S1. A neighbor joining tree of all sequences from Fungene's butyryl-CoA:acetate CoA-transferase (*but*) database (>93 % coverage to model; to ensure only full length sequences were considered). All *but* reference sequences with known function [10] group together in the section highlighted in green and apart from 4-hydroxybutyrate:butyryl CoA transferases (*4hbt*, highlighted in red). Several reference sequences from each group are indicated as stars (for *but* see Table S1, *4hbd*: *Clostridium klyuveri* (153955632), *C. tetani* (28210230), *Anaerostipes caccae*

(76096774) and *C. aminobutyricum* (188032706)). All sequences in the green section are considered probable *but* sequences in this study. For details about primer coverage see Table S1.

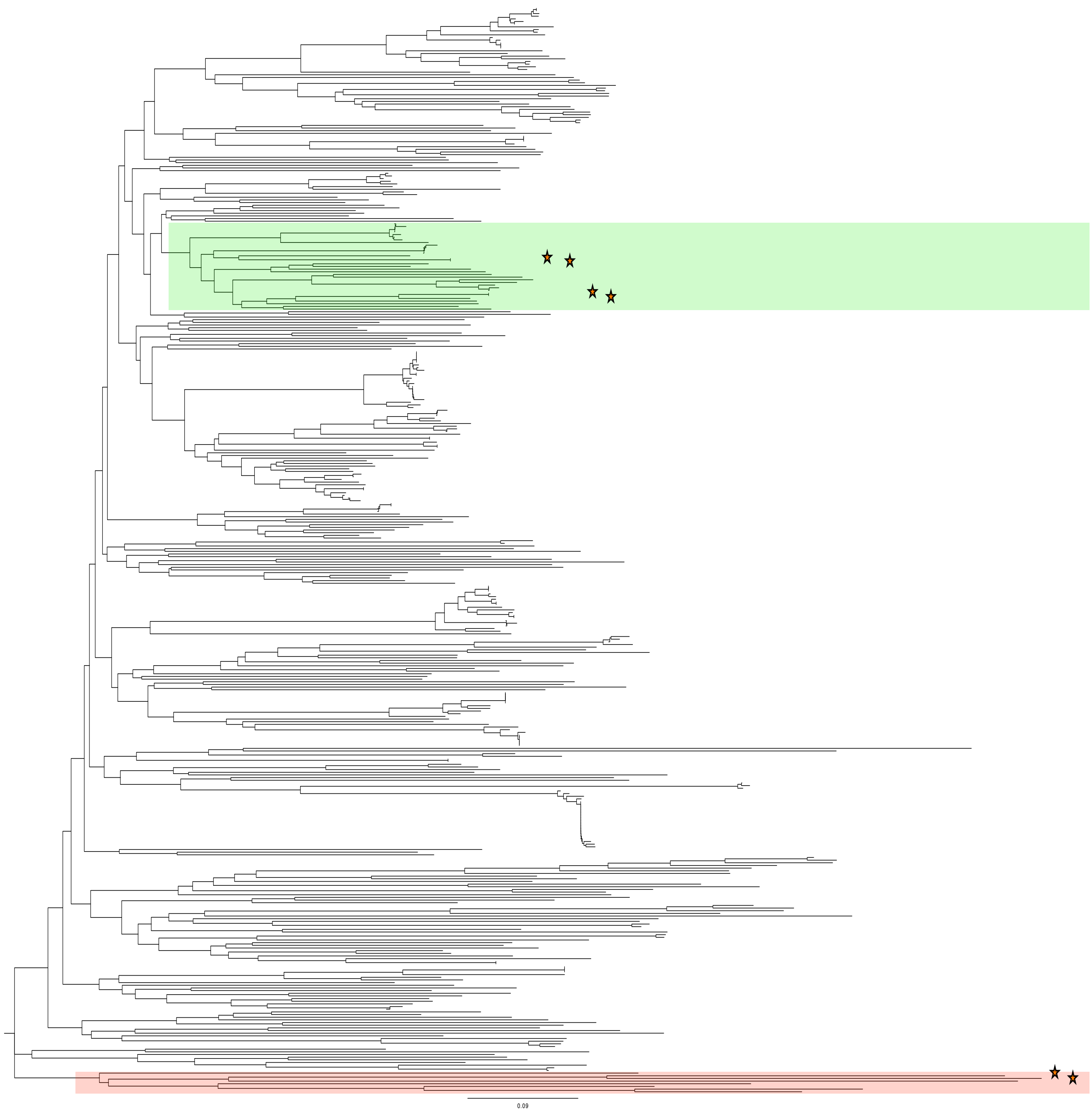

Figure S2. A neighbor-joining tree of all sequences from Fungene's butyrate kinase (*buk*) database (>93 % coverage to model; to ensure only full length sequences were considered). Eighty-eight percent of sequences are annotated as butyrate kinase and most sequences cluster apart from acetate kinase, a closely related gene (highlighted in red; two sequences with known acetate kinase function from *Bacillus subtilis* (405134) and *Escherichia coli* K-12 (67462089) were added to the tree (indicated as a star)). Only a few sequences have been verified

biochemically as butyrate kinases ([10], indicated as stars) and all clustered together in one group (highlighted in green). Primers were designed to target most of the sequences in the green block as well as many targets outside this group. For details see Table S2.

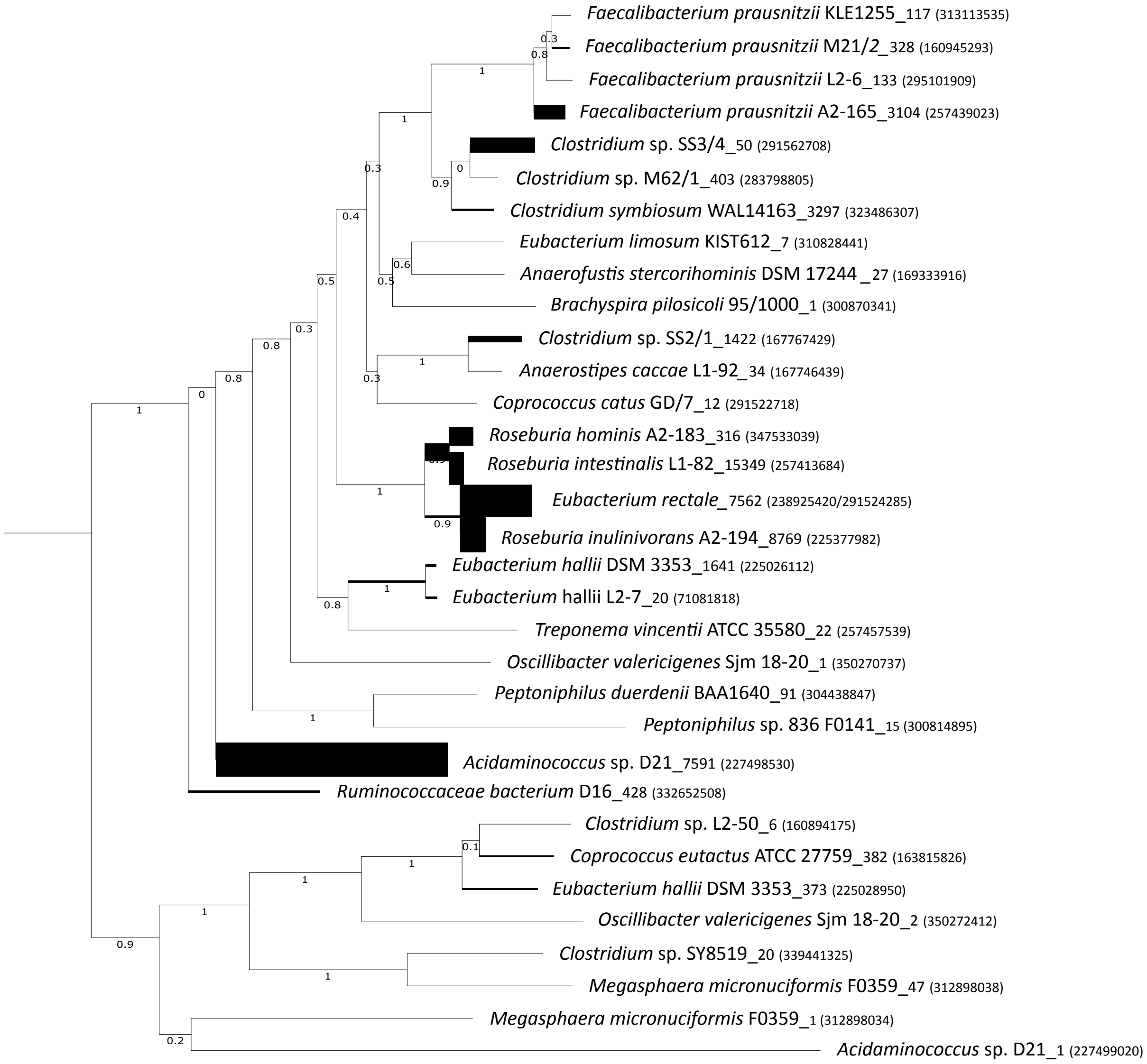

Figure S3. A maximum likelihood tree of FrameBot reference sequences for butyryl-CoA:acetate CoA-transferase (*but*) using PhyML [34]. Each amplicon sequence was placed onto this fixed reference tree using Pplacer [24] under maximum likelihood criteria. The height of each branch is proportional to the number of amplicons diverging from the tree along the branch. Bootstrap values are indicated.

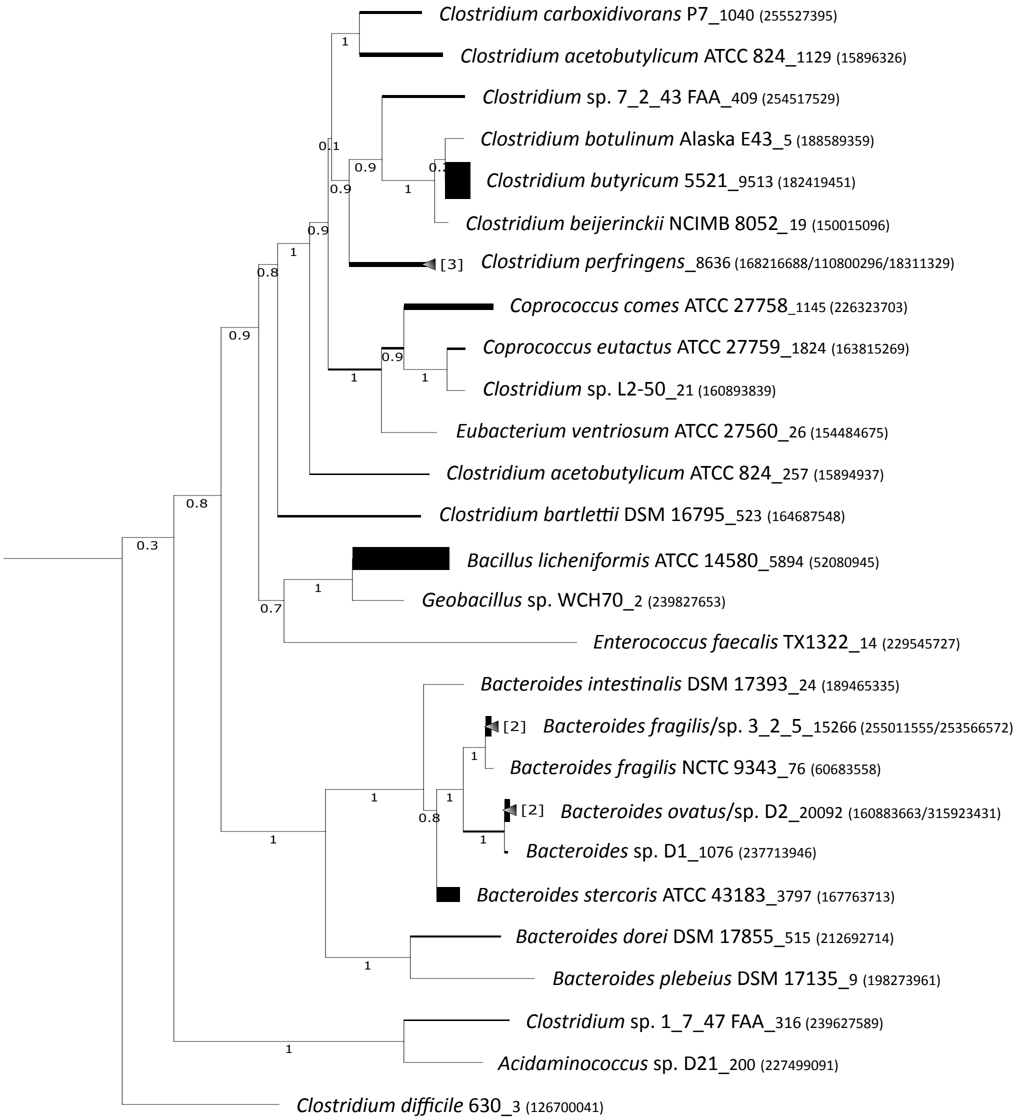

Figure S4. A maximum likelihood tree of FrameBot reference sequences for butyrate kinase (*buk*) using PhyML [34]. Each amplicon sequence was placed onto this fixed reference tree using Pplacer [24] under maximum likelihood criteria. The height of each branch is proportional to the number of amplicons diverging from the tree along the branch. Bootstrap values are indicated.

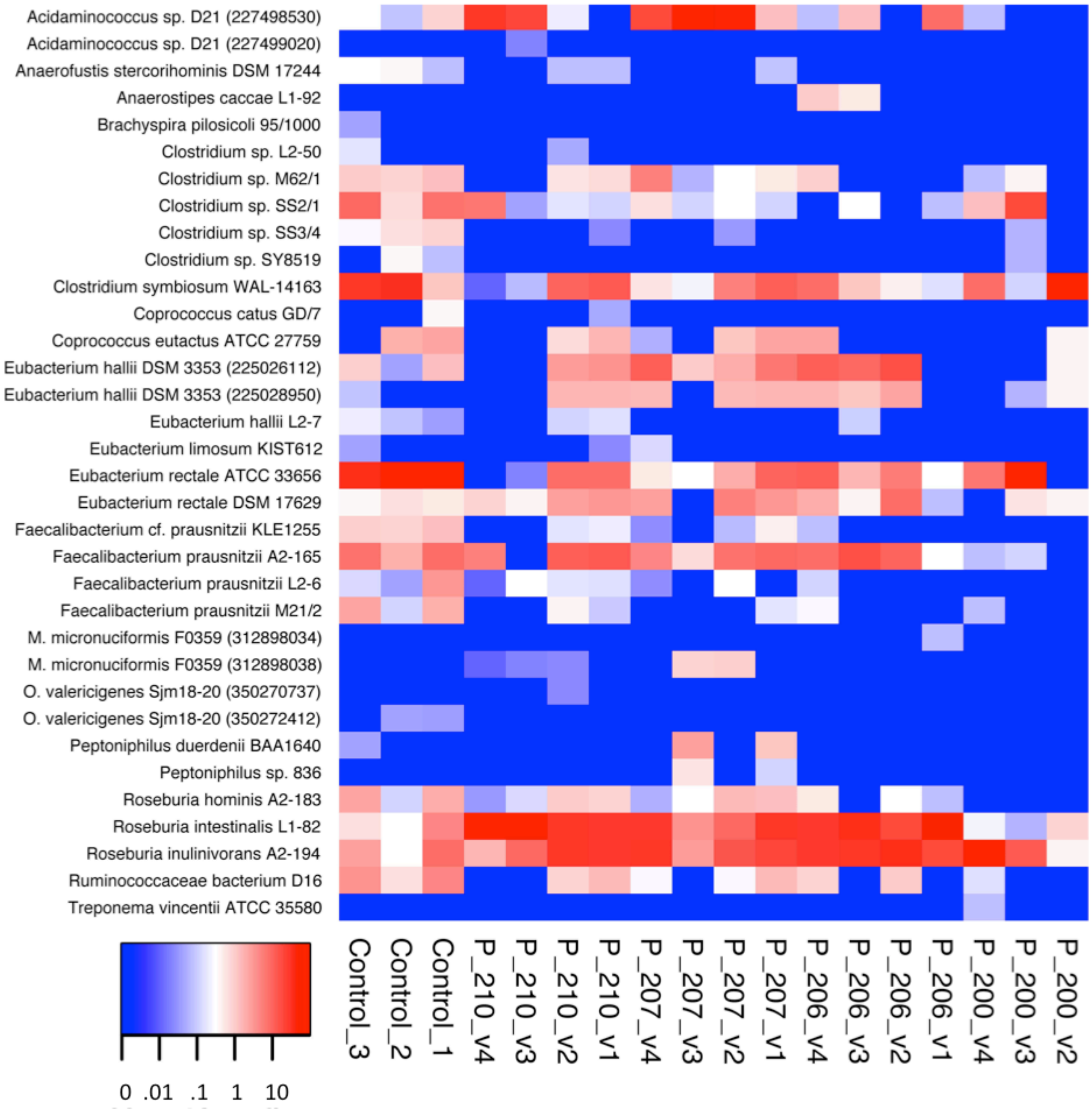

Figure S5. Pyrosequencing results of amplified butyryl-CoA:acetate-CoA transferase (*but*) sequences. Results are shown as percentage (log10) of total reads per sample.

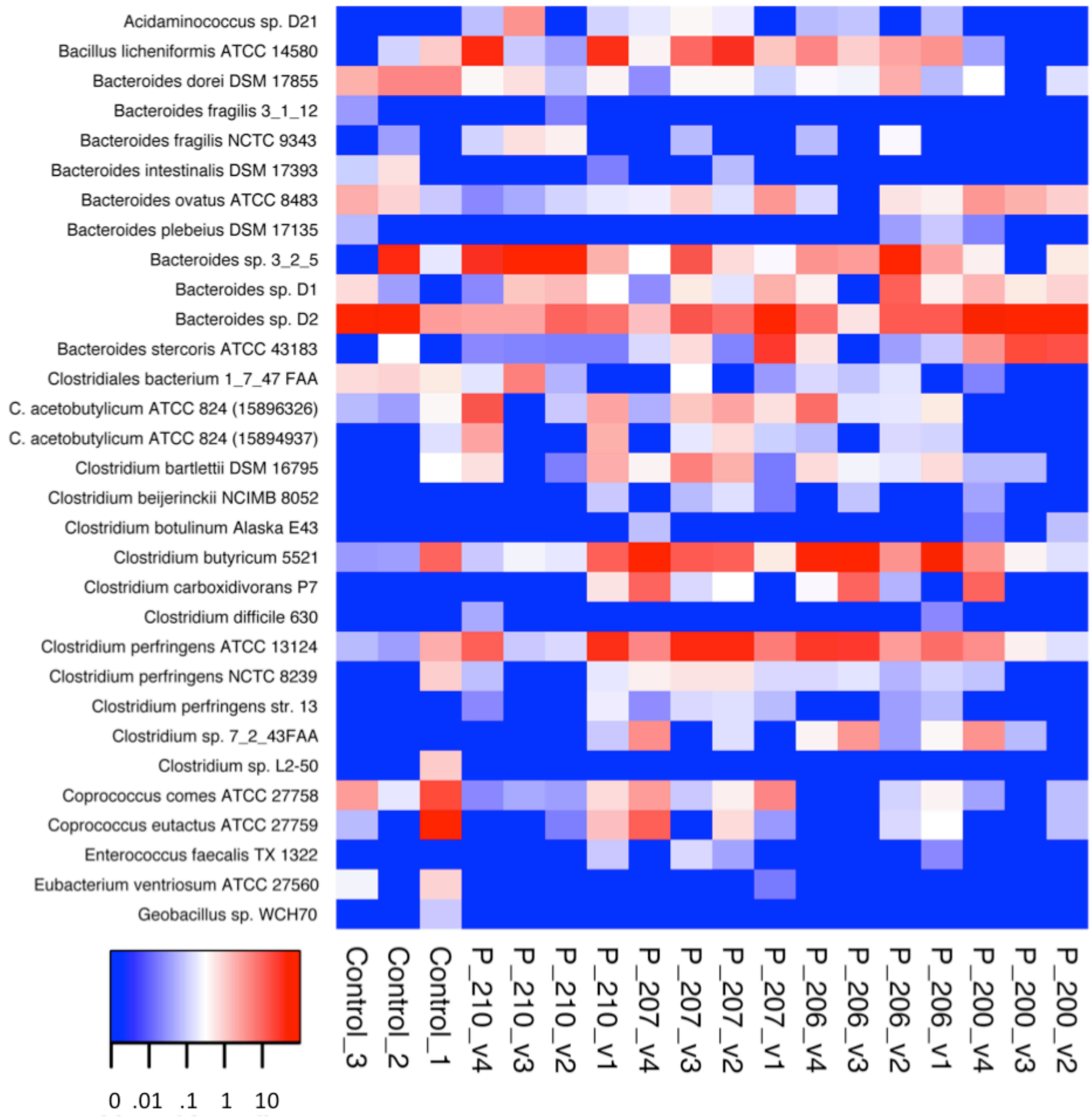

Figure S6. Pyrosequencing results of amplified butyrate kinase (*buk*) sequences. Results are shown as percentage (log10) of total reads per sample.

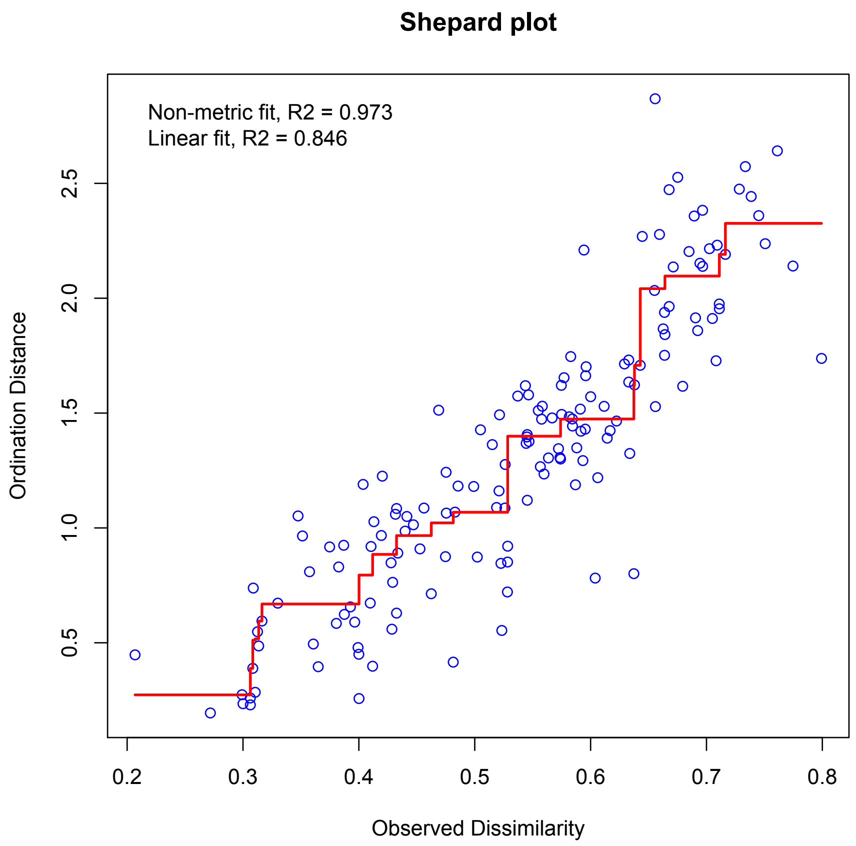

Figure S7. Shepard plot of the nonmetric multidimensional scaling (NMDS) analysis shown in Figure 3.

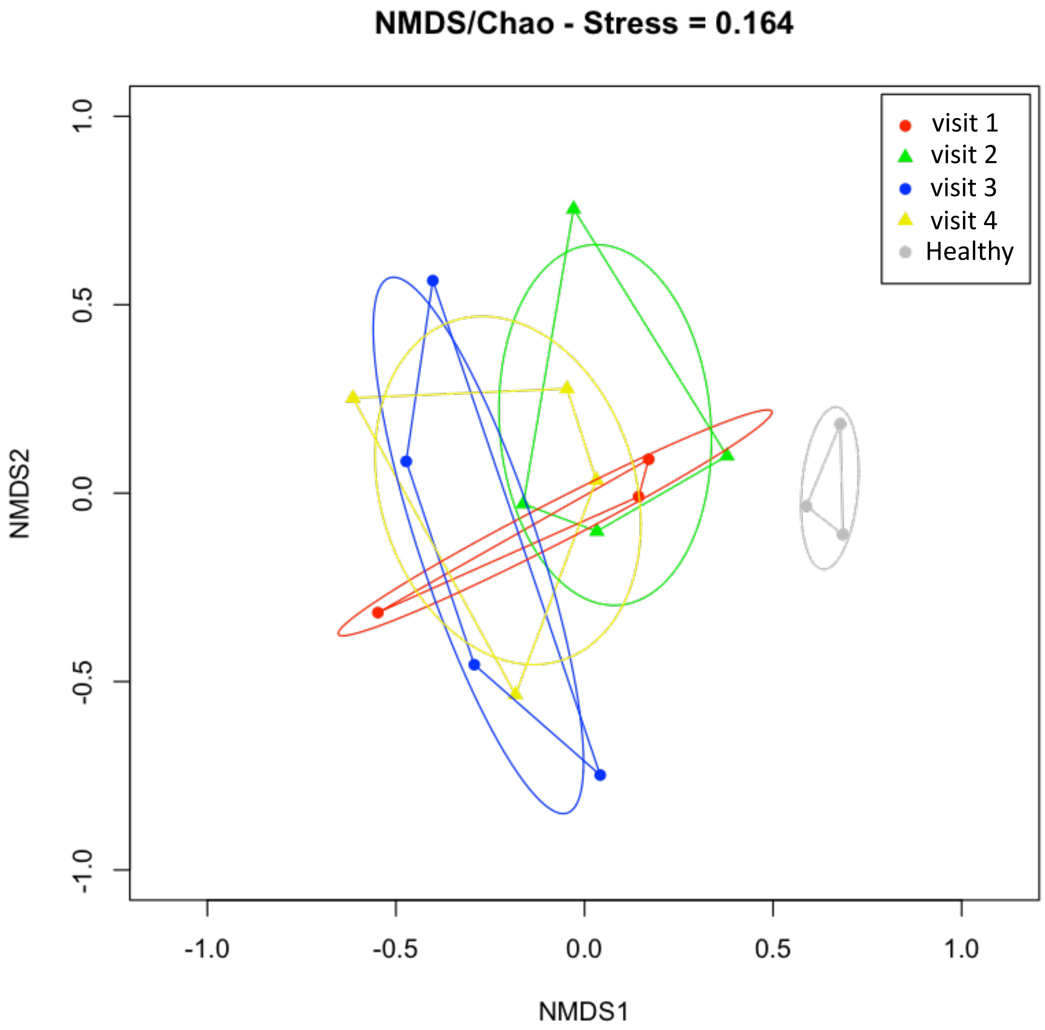

Figure S8. Nonmetric multidimensional scaling (NMDS) analysis of the total butyrate producing community - butyryl-CoA:acetate-CoA transferase (*but*) *a*nd butyrate kinase *(buk)* genes together - based on visits is shown. Ellipses represent the 95 % confidence interval on standard errors of means.
